# Pregnancy outcomes in C3 glomerulopathy: a retrospective review

**DOI:** 10.1186/s12882-025-04118-y

**Published:** 2025-05-14

**Authors:** Lauren O. Fergus, Meryl Waldman, Monica D. Hall, Lynn Vining, Jillian Hall, Tina Liu, Yuzhou Zhang, Patrick J. Walker, Richard J. H. Smith, Carla M. Nester

**Affiliations:** 1 University of Iowa Molecular Otolaryngology and Renal Research Laboratories, Iowa City, IA USA; 2 Kidney Disease Branch, National Institute of Diabetes and Digestive and Kidney Diseases, National Institutes of Health, Bethesda, MD USA; 3 Arkana Laboratories, Little Rock, AZ 72211 USA; 4 University of Iowa Hospitals and Clinics, Iowa City, IA USA; 5 Stead Family Children’s Hospital, Iowa City, IA USA

**Keywords:** C3 glomerulopathy, Pregnancy, Glomerular disease, Complement-mediated kidney disease, Women’s health

## Abstract

**Background:**

C3 Glomerulopathy (C3G) is an ultra-rare glomerular disease driven by dysregulation of the alternative pathway of complement. 30–50% of adult patients progress to end stage kidney disease (ESKD) within 10 years of diagnosis. Little is known of the impact of pregnancy on the natural history of C3G or whether a coincident diagnosis of C3G affects maternal-fetal outcomes.

**Methods:**

Female subjects from the University of Iowa’s C3G Natural History Study who met consensus biopsy criteria were included if they had at least one pregnancy and available renal/obstetric data. Assessed data included clinical history, kidney function tests, and complement tests to identify genetic and/or acquired drivers of complement dysregulation. Appropriate *t*-tests or *z*-tests were used to compare outcomes and clinical biomarker changes pre-/post-pregnancy. Nonlinear regression and relative risk were used to estimate risk for preeclampsia, premature delivery, and progression to ESKD.

**Results:**

Amongst mothers whose C3G presented before or during pregnancy (C3G + P), there were 37 pregnancies and 27 deliveries. Non-live birth outcomes impacted 10 C3G + P and included 5 spontaneous miscarriages, 1 stillbirth, 1 ectopic pregnancy, and 3 elective abortions. Twelve deliveries (44%) were premature, while 16 (59%) were associated with antepartum preeclampsia: an elevated risk when compared to healthy pregnancies and pregnancies of mothers with other glomerular diseases. Risk factors for complications included preexisting hypertension, an identified driver of complement dysregulation, and an eGFR prior to pregnancy of < 60 ml/min/1.73m^2^. These risk factors also predict progression to ESKD within 5 (5/32, 16%) and 10 years (6/32, 19%) following pregnancy. Compared to pre-pregnancy values, post-pregnancy serum creatinine levels trended upwards and eGFRs downwards, both by small but significant amounts. Individual pre-/post-pregnancy eGFRs were significantly worse in mothers who progressed to ESKD within 5–10 years of pregnancy.

**Conclusions:**

A C3G + P is associated with increased risk of preeclampsia and prematurity compared to healthy controls, but no excess risk of spontaneous miscarriage. A C3G + P was associated with a small but significant decrease in renal function as measured by change in creatinine and eGFR. The elevated risk of adverse renal and obstetric events supports the need for multidisciplinary care for expectant patients with C3G.

**Supplementary Information:**

The online version contains supplementary material available at 10.1186/s12882-025-04118-y.

## Introduction

Little is known about the outcomes for women who experience pregnancy in the setting of C3 Glomerulopathy (C3G). This knowledge gap reflects the ultra-rare nature of C3G and the relatively recent recognition that the disease includes much more than membranoproliferative glomerulonephritis type II (MPGN II) or Dense Deposit Disease (DDD) [1]. The latter is pertinent because, while there are older series of data describing the outcomes of pregnancy in all types of MPGN [2, 3], it is impossible to extract C3G-specific outcomes from these studies to apply to current practice. This paucity of data makes pregnancy counseling incomplete and presents difficulties for patients and physicians alike [4].

Chronic kidney disease as an entity has been associated with adverse outcomes in pregnancy, although some of these issues can be managed with prospective monitoring and counseling [5–7]. Larger cohorts of pregnancies in patients with generalized glomerular disease (IgA Nephropathy, Minimal Change Disease, etc.) also have mixed outcomes, but with consistent reporting of increased preterm deliveries and elevated preeclampsia risk [8–10]. C3G, in particular, presents a unique situation in the context of pregnancy due to its epidemiology and pathogenesis. With respect to the former, patients with C3G typically present in early adulthood [11], which may impact their ability to pursue pregnancy (due to use of teratogenic medication, counseling against pregnancy as a renal protective measure, etc.). And with respect to the latter, dysregulation of the alternative complement pathway is essential to the disease process of C3G. Complement hyperactivity has been implicated in pathological situations like preeclampsia and spontaneous miscarriage [12, 13], raising questions about the possible impact of underlying complement dysregulation in these two situations. Hypocomplementia, often seen in C3G, has also been linked to poor pregnancy outcomes in lupus nephritis patients, another complement activity related renal disorder [14, 15].

In this study, we surveyed data on females enrolled in the University of Iowa’s C3G Natural History Study and examined the course of their pregnancies, short-term events, and long-term renal outcomes. Our aim was to provide an objective description of the outcomes of pregnancy in patients with C3G, identify possible risk factors or conditions that may contribute to adverse events both to mother or baby during and after pregnancy, and produce data which nephrologists may use to counsel around pregnancy in the setting of C3G, better informing patients and their families of related risks.

## Materials & methods

The University of Iowa’s C3G Natural History Study includes both a retrospective arm spanning back to 1983 and a prospective arm enrolling since 2017. All enrolled subjects are diagnosed by a kidney biopsy consistent with the consensus definition of C3G [1] and each biopsy is interpreted by at least one pathologist and one nephrologist. Both pathologic subtypes of C3G (DDD and C3 Glomerulonephritis/C3GN), are included. Clinical data are managed using REDCap electronic data capture on site at the University of Iowa [16, 17]. To be included in the pregnancy cohort, an individual must have had a native kidney biopsy diagnosis of C3G, female sex, ≥ 15 years of age at time of data extraction, had ≥ 1 pregnancy, and have available both obstetric and renal data. Outcomes were compared to that of the healthy US population, as well as pregnancies of individuals with IgA Nephropathy (chosen due to similarity to C3G as another primary, proliferative glomerular disease) [18], a cohort of Individuals with generalized glomerular disease (minimal change disease, membranous nephropathy, etc.) [10], and historical data from studies describing pregnancy in MPGN Type I patients [3], which based on prior disease definition would have included patients with what we now refer to as immune complex glomerulonephritis (ICGN) *and* some types of C3GN (formerly MPGN Type I) [19].

Data were extracted through electronic medical records and patient interviews using standardized questions. Standard demographics and longitudinal renal and obstetrical clinical data including kidney biopsies, lab values, and data describing the health of mother and baby throughout the pregnancy and beyond were collected. Renal-related lab values were obtained, where available, up to three to six months before estimated conception and after termination/delivery to investigate effects of pregnancy on baseline renal health. Assessed data included systolic and diastolic blood pressures, serum creatinine (sCr), estimated glomerular filtration rate (eGFR), proteinuria as measured on urine dipstick and urine protein to creatinine ratio, weight, and complement C3. Complement biomarker and genetic profiles were collected through testing by the Molecular Otolaryngology and Renal Research Laboratories (MORL) to assess for drivers of complement dysregulation.

Standard definitions were used to describe events of pregnancy, as demonstrated in Table 1. Adverse events of pregnancy were defined in this study as all forms of pregnancy loss (besides elective abortion), the development of preeclampsia or eclampsia, premature delivery, or delivery of a low-birth-weight infant. Premature birth was categorized as delivery before 37 weeks gestation and a low-birth-weight infant was defined as weighing less than 5.5 lb. (or 2500 g - <10th percentile for gestational age) at delivery. Pregnancies were further categorized by whether they occurred before initial C3G presentation (P + C3G) or during/after initial C3G presentation (C3G + P). Pregnancy during initial C3G presentation was defined as new-onset hematuria at any point during the pregnancy, worsening proteinuria in the 1st trimester, or worsening serum creatinine throughout the course of the pregnancy, with disease confirmed by kidney biopsy after delivery/termination. Mothers were also identified by whether they had an identified driver of their C3G. A positive driver included a predicted pathogenic genetic variant, C3/C4/C5 nephritic factors, Factor B/Factor H autoantibodies, or a monoclonal immunoglobulin [20]. Genetic variants of unknown significance were not considered drivers of disease but are still reported. Additional information regarding genetic variants in the cohort is available in Supplemental Table 2.

**Table 1:** Study population definitions. Definitions of outcomes examined in this study. Genetic variants of unknown significance were reported in 10 of 32 mothers. For the purposes of our calculations, these variants were not considered driver of diseases and are included in Supplemental Table 2

Adverse Event of Pregnancy	All forms of pregnancy loss (besides elective abortion), the development of preeclampsia or eclampsia, premature delivery, or delivery of a low-birth-weight infant
Premature Birth	Delivery before 37 weeks’ gestation
Low Birth Weight	Infant weighing less than 5.5 lb. (or 2500 g - <10th percentile for gestational age) at time of delivery.
Positive Driver of Disease	Pathogenic genetic variant (as classified according to ACMG guidelines), C3/C4/C5 nephritic factors, Factor B/Factor H autoantibodies, or monoclonal proteins. [20].

### Statistical analysis

Means with standard deviation/95% confidence intervals, as well as medians with inter-quartile range, are reported where appropriate. Comparisons between pre-/post- pregnancy lab values use Wilcoxon matched-pairs signed rank test, while lab data comparisons between outcomes use the unpaired two-tailed *t*-test with Welch’s correction. Event prevalence is reported as percentages and compared with published U.S. population and relevant study values (95% confidence intervals reported when available) using the one-proportion and two-proportion two-tailed *z*-test. Spearman’s correlation was used to examine the relationship between variables. Outcomes were coded dichotomously as 0 = No event, 1 = Event, for inclusion in this analysis. Chi square with Fischer’s exact test was used to calculate relative risks. Non-linear regression was utilized to predict change in lab values pre/post pregnancy, while a receiver operating characteristic (ROC) curve was used to determine predictive cut-offs for progression to ESKD. Both 95% confidence intervals and p values were included in appropriate analyses. All statistical calculations were performed in GraphPad Prism 10 (GraphPad Software LLC, 2023, Boston, Massachusetts USA, www.graphpad.com). A p value of ≤ 0.05 was considered statistically significant.

## Results

**Fig. 1:**
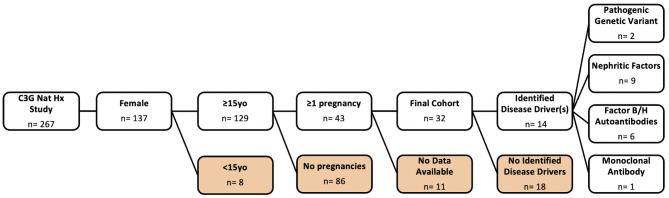
Cohort determination. Flowchart demonstrating cohort selection criteria, final sample size, and drivers of disease. Orange boxes represent exclusions. Of the four individuals with multiple drivers of disease, three had nephritic factors and Factor B/H autoantibodies, and one had a monoclonal immunoglobulin and Factor B/H autoantibodies

Of 43 women with ≥ 1 pregnancy identified in the database, 32 women (representing 79 pregnancies) had sufficient data to be included in the analysis (Fig. 1). Baseline characteristics are summarized in Table 2. All pregnancies occurred between 1965 and 2023, with 54% of the pregnancies having obstetrical records available for review. The majority had a diagnosis of C3GN (25/32, 78%), while 7/32 (22%) had a diagnosis of DDD, consistent with the typical distribution of C3G [21]. The mean maternal age in the cohort was 27y 4 m (SD ± 5y 8 m), with a range from 16 to 39 years old. The median number of lifetime pregnancies was 2, and ranged from 1 to 8 total pregnancies.

Drivers of disease were identified in 44% (14/32) of the cohort, and 13% (4/32) had more than one driver. Drivers included a pathogenic gene variant (2 patients; Supplemental Table 2), C3/C4/C5 nephritic factors (9 patients), Factor B or Factor H autoantibodies (6 patients), and a monoclonal immunoglobulin (1 patient). Over half (18/32, 56%) of the patients did not have an identified driver of disease. Additionally, 31% (10/32) had at least one genetic variant of unknown significance (VUS) (Supplemental Table 2).

**Table 2:** Cohort demographics. Demographic information and C3G disease characteristics of cohort

Characteristics	n = 32 (%) [SD]
Maternal Age	27y 4m [± 5y 8m]
Race/Ethnicity	
White, Non-Hispanic	24 (75%)
White, Hispanic	5 (16%)
Black	2 (6%)
Asian/Pacific Islander	1 (3%)
Native American	0 (0%)
Pathologic Diagnosis	
C3GN	25 (78%)
DDD	7 (22%)
Drivers of Disease	
Any Driver	14 (44%)
Pathogenic Genetic Variant	2 (6%)
Nephritic Factors	9 (28%)
Factor B/Factor H Autoantibodies	6 (19%)
Monoclonal Immunoglobulin	1 (3%)
None Identified	18 (56%)
Genetic Variants of Unknown Significance	10 (31%)

**Fig. 2:**
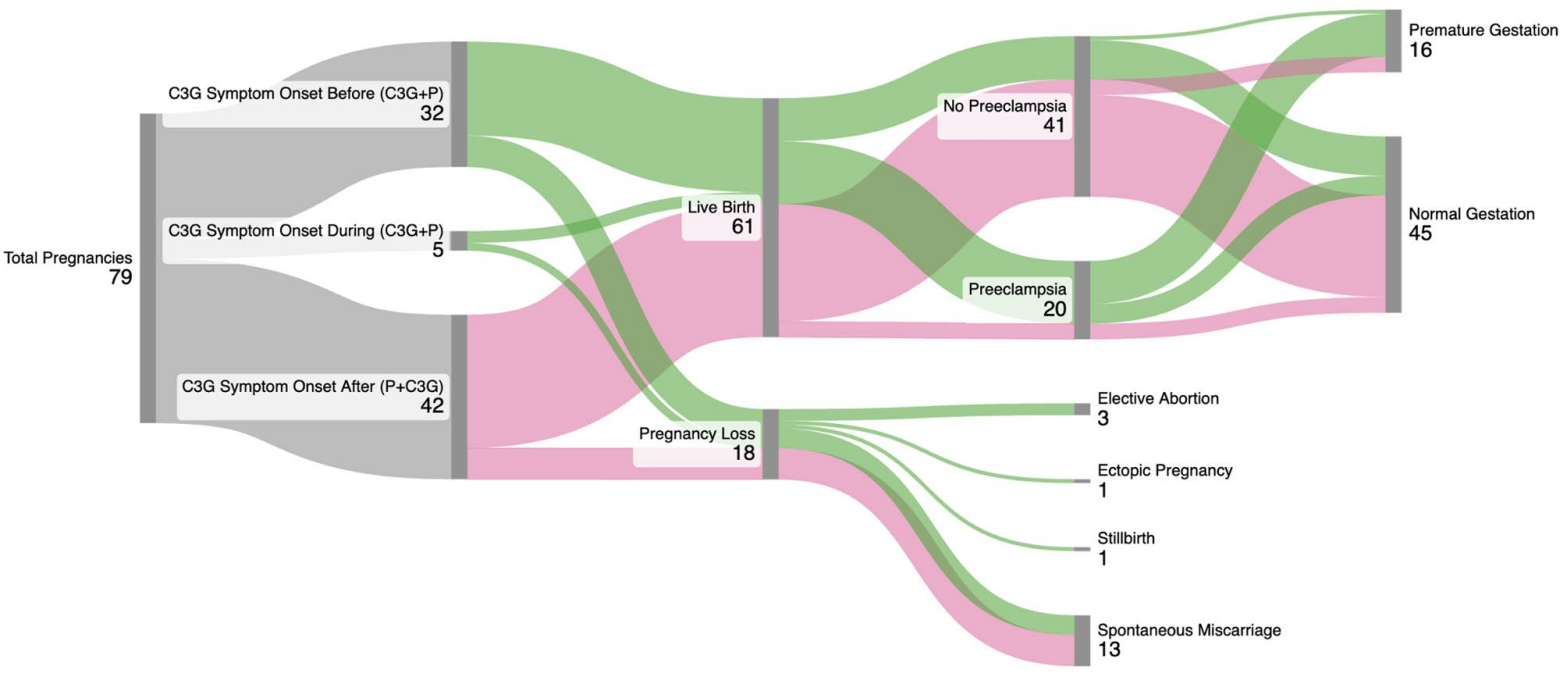
Pregnancy outcomes flowchart. Sankey diagram representing pregnancy outcomes of the cohort in relation to time of C3G diagnosis. Green flows represent C3G + P while pink flows represent P + C3G

Outcomes of pregnancy are characterized in Fig. 2; Table 3. Of 79 pregnancies, 47 (59%) occurred prior to the histologic diagnosis of C3G (P + C3G) and 32 (41%) occurred after histologic diagnosis (C3G + P). Although no one had a kidney biopsy during pregnancy, 5 (16%) patients’ initial symptoms of glomerulonephritis presented during this time period; in these cases, the diagnostic kidney biopsies occurred weeks to months after delivery or at termination of the pregnancy and these patients are included in the C3G + P group. Eighteen pregnancies (18/79, 23%, 8/42 P + C3G vs. 10/37 C3G + P) did not go to completion, usually due to spontaneous miscarriage prior to the 20th week of gestation (13/18, 8/42 P + C3G vs. 5/37 C3G + P). There were three elective abortions, a single ectopic pregnancy, and one stillbirth, all in the C3G + P cohort. Twenty deliveries (20/61, 33%, 4/42 P + C3G vs. 16/37 C3G + P) were complicated by preeclampsia and one delivery (1/61, 2%) in the C3G + P group was complicated by eclampsia at 35 weeks. HELLP syndrome was not diagnosed. 16/32 (50%) C3G + Ps had worsening of blood pressure during pregnancy and required anti-hypertensive therapy (labetalol or nifedipine); 10/16 (63%) individuals in this group developed subsequent preeclampsia. No P + C3Gs required anti-hypertensives. There were seven pregnancies total from two individuals with a pathogenic variant (see Supplemental Table 2). Two ended in spontaneous miscarriage, and one was terminated electively. Of the remaining four pregnancies, 2/4 were complicated by preeclampsia. There was no premature delivery or neonatal complications in this group.

**Table 3:** Pregnancy descriptions and outcomes. Pregnancy outcomes of the cohort and characteristics of delivered infants. P + C3G consists of pregnancies occurring before C3G presentation, while C3G + P consists of those occurring during or after C3G presentation

Outcomes		*n* = 79 pregnancies and 61 deliveries (%) [SD]	
**Timing of Diagnostic Biopsy**			
Before Pregnancy		47 (59%)	
During Pregnancy		0 (0%)	
After Pregnancy		32 (41%)	
**Mother’s Onset of C3G Symptoms**			
Before Pregnancy		32 (41%)	
During Pregnancy		5 (6%)	
After Pregnancy		42 (52%)	

	**Full Cohort** *n* = 79 pregnancies and 61 deliveries	**P + C3G** *n* = 42 pregnancies and 34 deliveries	**C3G + P** *n* = 37 pregnancies and 27 deliveries
**Gestation**			
Mean Gestational Age	37w 6d (± 2w 4d)	38w 5d (± 2w 0d)	36w 4d (± 2w 6d)
Median Gestational Age	39w 0d	40w 0d	37w 0d
Prematurity (< 37 weeks)	17 (28%)	4 (12%)	12 (44%)
**Birth Weight**			
Mean Birth Weight	6 lb. 11 oz (± 1 lb. 10 oz)	7 lb. 6 oz (± 1 lb. 5 oz)	5 lb. 14 oz (± 1 lb. 7 oz)
Median Birth Weight	6 lb. 13 oz	7 lb. 7 oz	6 lb. 1 oz
Low Birth Weight (< 5.5 lbs.)	12 (20%)	1 (3%)	11 (41%)
**Pregnancy Loss**	18 (23%)	8 (19%)	10 (27%)
Spontaneous Miscarriage	13 (72%)	8 (100%)	5 (50%)
Elective Abortion	3 (17%)	0 (0%)	3 (30%)
Ectopic Pregnancy	1 (6%)	0 (0%)	1 (10%)
Stillbirth	1 (6%)	0 (0%)	1 (10%)
**Cesarean Section**	18 (30%)	6 (18%)	12 (44%)
**Preeclampsia**	20 (33%)	4 (12%)	16 (59%)
**Eclampsia**	1 (2%)	0 (0%)	1 (4%)
**HELLP Syndrome**	0 (0%)	0 (0%)	0 (0%)
**Maternal Death**	0 (0%)	0 (0%)	0 (0%)
**Neonatal Health Issues**	5 (8%)	0 (0%)	5 (19%)
**Neonatal Death**	0 (0%)	0 (0%)	0 (0%)
**Gravidity**			
Primiparous	32 (41%)	15 (36%)	17 (46%)
Multiparous	47 (59%)	27 (64%)	20 (54%)

22/79 pregnancies had prior exposure to immunosuppression, including corticosteroids, mycophenolate mofetil, tacrolimus, rituximab, azathioprine, and eculizumab. Immunosuppression was stopped before pregnancy in 17 of these 22 pregnancies. In the 5 pregnancies with concurrent immunosuppressant use, 3 were terminated electively due to teratogenicity concerns, and eculizumab was utilized in the remaining 2. The first continued eculizumab throughout pregnancy with no major obstetric or renal complications post-delivery. In the second pregnancy, eculizumab was initiated at 28 weeks’ gestation as a renal-protective measure. Ultimately, the mother developed preeclampsia, delivering prematurely at 34 weeks gestation and progressing to ESRD within six months.

**Fig. 3:**
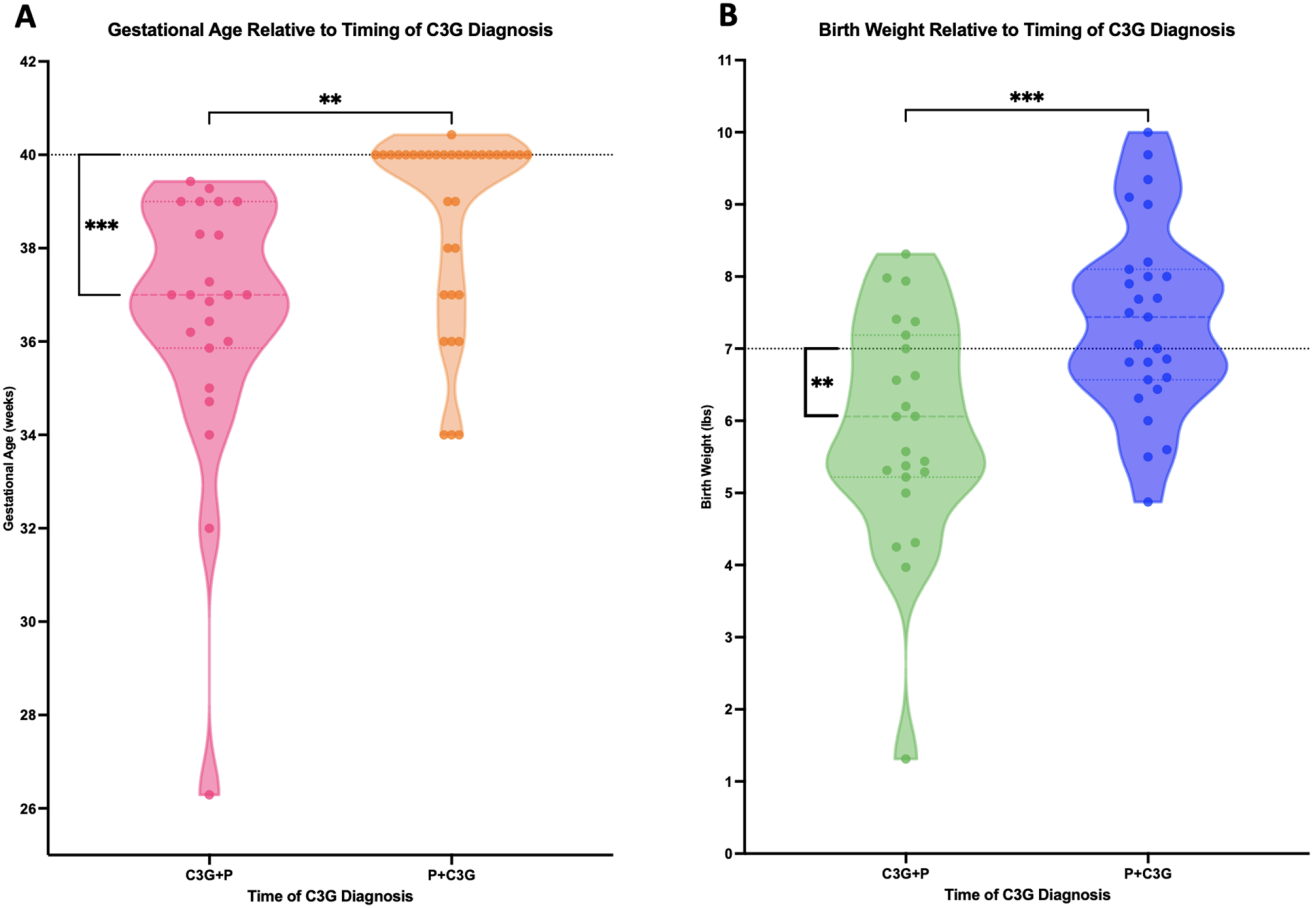
Gestational age and birth weight analyses. Comparisons of gestational age (**A**) and birth weight (**B**) to normal values and within the cohort by timing of C3G diagnosis (C3G + P vs. P + C3G). These groups were compared using unpaired t-test with Welch’s correction, represented by asterisks between the violin plots. Standard “normal” values of 40 weeks and 7 lb. 0 oz., respectively, were also compared against C3G + P using one-sample t-test, represented by asterisks between the violin plot and dashed lines

Gestation and birth weight analyses are demonstrated in Fig. 3. Seventeen infants (17/61, 28%) were premature. Gestation ranged from 26w 2d to full term, with a mean of 37w 6d (SD ± 2w 4d) and median of 39w 0d in the full cohort. As compared to an expected full-term gestation of between 39 weeks, 0 days and 40 weeks 6 days [22], deliveries from C3G + P mothers occurred approximately 3 weeks earlier (Mean 36w 4d, 95% CI 35w 2d – 37w 4d, p = < 0.001). Mean duration of gestation was lower in the C3G + P cohort as compared to the P + C3G cohort (36w 4d vs. 38w 5d respectively, 95% CI, 0w 6d – -3w 4d, *p* = 0.0012).

Birth weights in the full cohort ranged from 1 lb. 5 oz to 10 lb. 0 oz, with a mean of 6 lb. 11 oz (SD ± 1 lb. 10 oz) and a median of 6 lb. 13 oz; 12 infants (12/61, 20%) were considered low birth weight at delivery. Mean birth weight for C3G + P deliveries was 5 lb. 14 oz. (95% CI 5 lb. 3 oz. – 6 lb. 9 oz.) as compared to 7 lb. 6 oz. (95% CI 6 lb. 14 oz. – 7 lb. 15 oz.) for P + C3G deliveries (*p* = 0.003) (Fig. 3). 18/61 (30%, 6/34 P + C3G vs. 12/27 C3G + P) of the deliveries were from cesarean Sect. 5/61 (8%) of the infants delivered had neonatal health issues requiring NICU hospitalization, all of which originated from C3G + P (Supplemental Table 3). There were no cases of maternal or neonatal death in either group.

### Selected comparisons

#### Risk factors for adverse events

Risk factors for adverse events of pregnancy were explored, as shown in Table 4. In our complete C3G cohort, preexisting hypertension was a risk factor for prematurity (RR 1.88; 95% CI 1.22–3.45, *p* = 0.004), a low-birth weight infant (RR 1.45; 95% CI 1.07–2.31, *p* = 0.03), and preeclampsia (RR 2.44; 95% CI 1.40–5.06, *p* < 0.001). Several of these associations are also observed within the healthy population (prematurity: RR 2.7, 95% CI 1.9–3.8; preeclampsia: RR 7.7, 95% CI 5.7–10.1 [23]). However, unique to our population was a preexisting C3G diagnosis (C3G + P), which was associated with the same adverse events: prematurity (RR 1.49; 95% CI 1.07–2.33, *p* = 0.03), a low-birth weight infant (RR 1.51; 95% CI 1.14–2.28, *p* = 0.006), and preeclampsia (RR 1.55; 95% CI 1.17–2.25, *p* = 0.003). Additionally, a preexisting eGFR < 60 ml/min was associated with both a risk for premature delivery (RR 4.20; 95% CI 1.15–23.63, *p* = 0.05) and progression to ESKD within 10 years of pregnancy (RR 2.20; 95% CI 1.12–6.12, *p* = 0.03). The identification of a driver of complement dysregulation in C3G mothers was also a risk factor for preeclampsia (RR 1.68; 95% CI 1.08–2.99, *p* = 0.02). There were no associations with particular drivers with any of the adverse outcomes. Also of note, preeclampsia occurred in 11/41 (27%) deliveries without a known driver of disease. Advanced maternal age, primigravidity, significant proteinuria, or carrying a diagnosis of DDD as opposed to C3GN (and vice versa) were not independent risk factors (Table 4). Both a C3G diagnosis (RR 1.28; 95% CI 1.28–1.36, *p* = 0.006) and identified driver of disease (RR 3.55; 95% CI 1.11–19.68, *p* = 0.04) was associated with an increased risk for neonatal health issues, which included pneumothorax, neonatal stroke, and acute renal failure due to placental transfer of a maternal monoclonal immunoglobulin. These events are further detailed in Supplemental Table 3. Complete correlation data is demonstrated in Supplemental Fig. 2.

**Table 4:** Risk factors of adverse events during/after pregnancy. Relative risks of adverse events of pregnancy and other outcomes with selected maternal and C3G characteristics. P values and relative risks calculated with Chi-square and Fischer’s Exact Tests. Preexisting proteinuria was defined as a urine protein/creatinine ratio of ≥ 1.0

	Prematurity		Low birth weight		Preeclampsia		Spontaneous miscarriage		Health issues of neonate		ESKD within 10y of pregnancy	
	Relative risk (95% CI)	*P* value	Relative risk (95% CI)	*P* value	Relative risk (95% CI)	*P* value	Relative risk (95% CI)	*P* value	Relative risk (95% CI)	*P* value	Relative risk (95% CI)	*P* value
Advanced Maternal Age	0.69 (0.55–1.08)	0.17	0.78 (0.65–1.22)	0.33	0.93 (0.65–1.89)	> 0.99	1.05 (0.84–1.73)	0.67	0.91 (0.80–1.41)	> 0.99	1.06 (0.86–1.75)	0.65
Preexisting Hypertension	1.88 (1.22–3.45)	**0.004**	1.45 (1.07–2.31)	**0.03**	2.44 (1.40–5.06)	**< 0.001**	0.77 (0.72–1.10)	0.32	1.16 (0.97–1.62)	0.13	1.23 (0.98–1.73)	0.09
Preexisting eGFR < 60 ml/min	4.20 (1.15–23.63)	**0.05**	1.40 (0.73–3.85)	0.63	2.70 (0.67–15.60)	0.35	0.96 (0.80–1.42)	> 0.99	0.96 (0.66–1.88)	> 0.99	2.20 (1.12–6.12)	**0.03**
Preexisting Proteinuria	0.73 (0.25–1.76)	0.65	2.06 (0.97–4.59)	0.15	1.71 (0.48–5.89)	0.62	1.07 (0.72–1.90)	> 0.99	1.33 (0.83–1.81)	0.26	1.36 (0.80–2.92)	0.28
**DDD** vs. C3GN	0.80 (0.61–1.16)	0.31	0.90 (0.72–1.29)	0.71	1.13 (0.86–1.66)	0.40	1.21 (0.97–1.71)	0.17	1.19 (0.99–1.75)	0.09	0.84 (0.70–1.04)	0.16
Identified Disease Driver	1.07 (0.73–1.78)	0.77	1.39 (0.87–2.79)	0.31	1.68 (1.08–2.99)	**0.02**	1.06 (0.73–1.88)	> 0.99	3.55 (1.11–19.68)	**0.04**	1.32 (0.84–2.65)	0.34
C3G Dx **Before** vs. After Pregnancy	1.49 (1.07–2.33)	**0.03**	1.51 (1.14–2.28)	**0.006**	1.55 (1.17–2.25)	**0.003**	0.92 (0.75–1.15)	0.54	1.28 (1.28–1.36)	**0.006**	1.17 (0.95–1.53)	0.20
Primigravida	1.35 (0.99–1.99)	0.08	0.99 (0.76–1.33)	> 0.99	1.07 (0.82–1.45)	0.79	0.92 (0.75–1.15)	0.54	1.07 (0.90–1.36)	0.64	1.01 (0.83–1.28)	> 0.99

### C3G vs. a healthy population/iga nephropathy/mpgn/curegn cohort

The incidence of several adverse events of pregnancy associated with the diagnosis of C3G (C3G + P) were compared to several cohorts, as demonstrated in Table 5. These included published rates in a healthy cohort (US population data) [24–26], another primary glomerular disease (IgA Nephropathy) [18], a historical classification that included C3GN and ICGN (MPGN Type 1; limited data) [3], and the post-diagnosis subset of the CureGN cohort, consisting of patients with generalized glomerular disease including minimal change disease (MCD), membranous nephropathy, FSGS, IgA nephropathy or vasculitis [10]. Reported data consist of only pregnancies occurring during or after initial C3G presentation (C3G + P). The biggest difference was seen with preeclampsia, which occurred in the C3G cohort in 16 deliveries (16/27, 59%; 95% CI 39–79%), a significant increase as compared to healthy controls (4% incidence; p = < 0.001), patients with IgA nephropathy (7% incidence, 95% CI 5–11%; p = < 0.001), and MPGN Type 1 deliveries “complicated by hypertension” (31% incidence, 95% CI 19–43%; *p* = 0.01). This rate was similar to the prevalence of “complicated pregnancies” in the CureGN cohort (48% incidence, *p* = 0.36), which included one of at least of the following: worsening blood pressure, worsening kidney function, increased proteinuria, preeclampsia, eclampsia, or HELLP.

Prematurity was also more frequent in the C3G cohort as compared to healthy controls and the IgA nephropathy cohort (C3G vs. healthy controls: 44% (95% CI 24–64%) vs. 11%, *p* < 0.001; C3G vs. IgA nephropathy: 27% (44% CI 24–64%) vs. 8% (95% CI 6–13%), *p* < 0.001). The rate of prematurity was similar between C3G and MPGN Type 1 cohorts (27%, 95% CI 16–38%) vs. 29% (95% CI 18–41%), *p* = 0.17) and the CureGN cohort (25%, *p* = 0.09). There was no difference in rates of spontaneous miscarriage across groups (C3G vs. healthy controls, *p* = 0.42; C3G vs. IgA nephropathy, *p* = 0.87; C3G vs. MPGN Type I, *p* = 0.23, C3G vs. CureGN, *p* = 0.56).

**Table 5:** Incidence of adverse events in C3G+P cohort compared to healthy pregnancies and other glomerular diseases. Incidence rates of adverse events in C3G + P, compared to that of the healthy population and pregnancies of individuals with MPGN Type 1, IgA Nephropathy, and generalized glomerular disease (CureGN). CureGN cohort is pregnancies occurring after diagnosis of minimal change disease (MCD), membranous nephropathy, FSGS and IgA nephropathy or vasculitis. “Complicated pregnancies” included at least one of the following: worsening blood pressure, worsening kidney function, increased proteinuria, preeclampsia, eclampsia, or HELLP. P-values were calculated using two-proportion two-tailed z-test

Outcomes	C3G + *P* Cohort Prevalence (95% CI)	Healthy Pregnancy		MPGN Type 1 [3]		IgA Nephropathy [18]		CureGN Cohort [10]	
		Prevalence	*P* value	Prevalence (95% CI)	*P* value	Prevalence (95% CI)	*P* value	Prevalence	*P* value
Prematurity (< 37 weeks)	44% (24–64%)	11% (CDC 2021 [24])	**< 0.001**	29% (18–41%)	0.17	8% (6–13%)	**< 0.001**	25%	0.09
Spontaneous Miscarriage	14% (2–25%)	10% (ACOG 2018 [25])	0.42	24% (13–35%)	0.23	15% (11–20%)	0.87	10%	0.56
Preeclampsia	59% (39–79%)	4% (JAMA 2017 [26])	**< 0.001**	31% “complicated by HTN” (19–43%)	**0.01**	7% (5–11%)	**< 0.001**	48% “complicated pregnancies”	0.36

### Post-pregnancy renal disease

**Fig. 4:**
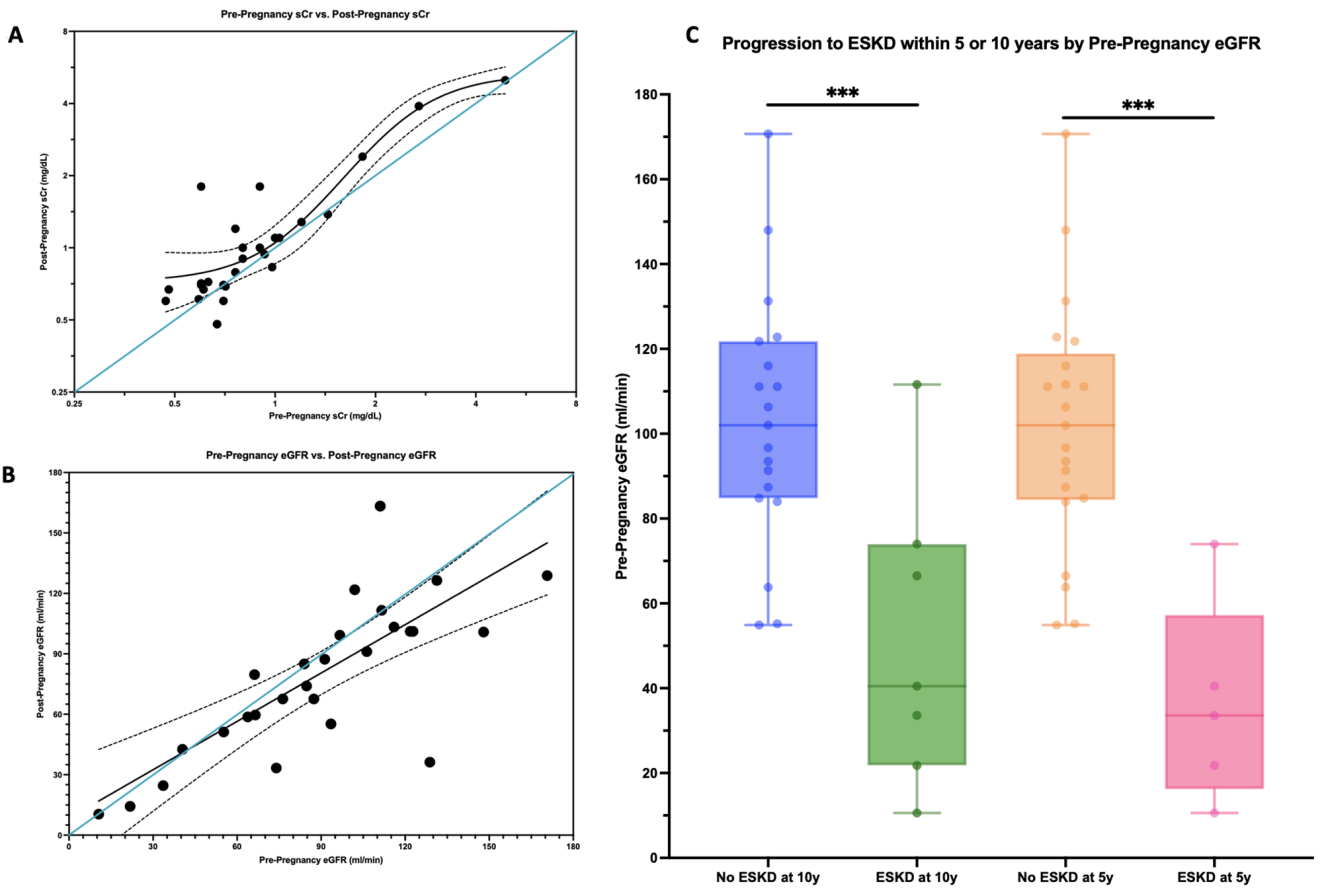
Determining risk of short and long-term disease progression in C3G + P cohort. Predicted progression of renal disease in association with C3G + P. In **A** and **B**, solid blue lines represent 1:1 pre-post values, or no change in lab values before and after pregnancy, while dashed lines represent 95% confidence bands of each line fit. Black dots represent individual pre-post values. Nonlinear regression was used (sigmoidal: **A**, linear: **B**). In **C**, unpaired t-test was used to compare mean pre-pregnancy eGFR’s in those who did and did not progress to ESKD within 5 and 10 years of pregnancy, with p-values represented as asterisks

We investigated whether C3G + P led to short- or long-term changes in kidney function as measured by eGFR and sCr. Additional data regarding changes in other lab values is available in Supplemental Fig. 1. 5/32 (16%) mothers progressed to ESKD within 5 years of a pregnancy, and 6/32 (19%) within 10 years. The change in eGFR and sCr in the months before and after pregnancy was modeled and is shown in Fig. 4. Change in sCr had a sigmoidal fit (R^2^ = 0.92), with only increases predicted post-pregnancy. For example, if pre-pregnancy sCr is at the upper limit of normal at 1.30 mg/dL, sCr is predicted to increase to 1.49 (95% CI 1.26–1.72). The change is eGFR was similar with a linear fit (R^2^ = 0.67) and predicts a decrease of 12% in post-pregnancy eGFR (95% CI -38 - +14%).

Patients who progressed to ESKD within 5 or 10 years of pregnancy had a mean pre-pregnancy eGFR of 36 ml/min (p = < 0.001) or 51 ml/min (*p* = 0.001) as compared to patients who retained native kidney function (mean eGFR ≥ 100 ml/min in both comparisons) (Fig. 4). There was no significant difference in patients who progressed to ESKD in 5 years vs. 10 years (*p* = 0.43). Beginning pregnancy with an eGFR of < 80 ml/min was associated with a likelihood ratio of progressing to ESKD within 5 or 10 years of 5.25 (ROC = 0.96, *p* = 0.04) or 5.43 (ROC = 0.86, *p* = 0.03) respectively.

## Discussion

One of the greatest strengths of this study is its relative size for this rare patient population. It is the largest cohort examining pregnancies in a well-defined, isolated C3G cohort. It is also one of the first studies examining pregnancy outcomes in C3G mothers at a scale larger than a case study, casting a much wider net of surveillance for what complications may occur in the context of pregnancy. Further, as a subset of the larger University of Iowa’s C3G Natural History Study, this study benefits from longitudinal data collection, allowing us the ability to evaluate the long-term changes associated with pregnancy as we continue to follow these women prospectively. This study also presents the opportunity to observe associations of pregnancy complications with disease characteristics of an individual’s C3G, including the drivers of disease and clinical lab correlates.

There are several limitations, the first being the utilization of patient survey as means of collecting general information on their pregnancies. All patient survey data were correlated with EMR data where available as a validation measure, yet some bias may be introduced by starting first with survey data. As the majority of our cohort are White individuals, both Hispanic and Non-Hispanic (consistent with this disease in general),– there are few conclusions that may be made for alternative ethnic backgrounds [21]. There was a lack of available data to describe complement activity during pregnancy in the context of C3G, which could play a role in disease presentation, progression, or alter the risk of adverse events, as has been reported in case studies [27].

We are additionally limited by a lack of renal data for pregnancies occurring before symptoms of glomerulonephritis and/or diagnostic C3G biopsy (P + C3G). It is unknown whether these patients had quiescent glomerular disease that was further worsened by pregnancy, leading to its eventual diagnosis, whether events like preeclampsia led to misdiagnosis of new onset glomerular disease, or if these pregnancies occurred in the absence of any disease at all and presentation of glomerular disease was triggered by a separate process. Similarly, acuity and chronicity histology data were not reviewed for this study, (due to lack of available data) which may have informed the GFR status of a given mother. Finally, individuals who are part of the C3G Natural History Study may not reflect the general patient population of C3G, primarily due to the heterogeneity of the disease.

One of the main challenges of pregnancy in women with C3G is distinguishing the early markers of preeclampsia from native kidney disease. Several manuscripts suggest the use of elevated Bb or C3a as an early marker of preeclampsia, both of which may be elevated in the nonpregnant C3G patient [28–30]. Further investigation into the predictive power of these proteins and other complement biomarkers in the C3G patient population could reveal patterns with the elevated risk of preeclampsia in this group and could be of predictive use in high-risk pregnancy counseling. Another suggested biomarker, the ratio of soluble fms-like tyrosine kinase 1 (sFlt-1) to placental growth factor (PlGF), has been reported as elevated in pregnant women before clinical onset of preeclampsia and eclampsia [31, 32]. If the same holds true in pregnant individuals with C3G, it could be of great clinical use, allowing for prophylactic measures to be taken and avoidance of less specific early detection methods that may have overlap between preeclampsia and native kidney disease.

Finally, as new complement inhibitors progress closer to availability for use in C3G, there may exist some clinical applications for their use in pregnancy. Treatment with eculizumab during pregnancy is considered relatively safe and has demonstrated some benefit in pregnancies complicated by paroxysmal nocturnal hemoglobinuria (PNH) and atypical hemolytic-uremic syndrome (aHUS), although its use is not without drawbacks and is not approved for pregnant C3G women [33–35]. Eculizumab (although with mixed efficacy in treating C3G [36–37]) and alternative pathway complement inhibitors, when or if more safety data is available in pregnant women, may have some benefit in this population, although more testing and research will be required.

## Conclusions

We present a comprehensive look at pregnancy in the context of C3 Glomerulopathy (C3G + P), including its obstetric and renal outcomes. C3G + P were at an increased risk of several adverse pregnancy events such as preeclampsia and premature delivery when compared to the healthy population and several other glomerular diseases, with no increased risk for spontaneous miscarriage. This study predicts a small, but notable decrease in kidney function in the C3G mother after pregnancy, similar to other glomerular diseases, and reveals significant differences in the pre-pregnancy eGFR’s of patients which progressed to ESKD. These data suggest that the true risks of pregnancy in individuals with C3G may have been overestimated historically. Our cohort experienced no maternal or neonatal deaths, with no incidences of HELLP syndrome, and only a single case of eclampsia. Although the risk of particular adverse events remains elevated in this patient population, it is comparable to the risk associated with other chronic kidney diseases and is manageable with proper surveillance and planning [38]. Of note, all three cases of elective abortion in our cohort cited the use of teratogenic medications (specifically mycophenolate mofetil and enalapril) as a contributing factor towards their decision to terminate the pregnancies, highlighting the continued need for education and patient-provider discussions regarding pregnancy within the context of C3G. These data provide a preliminary maternal-risk profile for C3G patients who are considering pregnancy and allow for certain risk scenarios to be considered and managed prospectively. Ultimately, the elevated chances of adverse events support the need for multidisciplinary pre- and peri-pregnancy care involving obstetrics, nephrology, and pediatrics as a mechanism for improving outcome of both mother and baby [4].

## Electronic supplementary material

Below is the link to the electronic supplementary material.

Supplementary Material 1

Supplementary Material 2

Supplementary Material 3

## Data Availability

All data generated or analyzed during this study are included in this published article and its supplementary information files.

## Declarations

### Dual publication

An abstract of a previous version of this work was presented as a poster at the American Society of Nephrology’s Kidney Week in 2021 [39]; the scope and size of the project has since been significantly expanded.

## Abbreviations

C3G: C3 Glomerulopathy
DDD: Dense Deposit Disease
C3GN: C3 Glomerulonephritis
MPGN: Membranoproliferative Glomerulonephritis
ICGN: Immune Complex Glomerulonephritis
eGFR: Estimated Glomerular Filtration Rate
sCr: Serum Creatinine
ESKD: End Stage Kidney Disease
C3G + P: Pregnancies occurring during or after initial C3G presentation
P + C3G: Pregnancies occurring before initial C3G presentation
