# Supplementary material for: Pregnancy outcomes in C3 glomerulopathy: a retrospective review
Source: BMC Nephrol. 2025 May 14;26:238. doi: 10.1186/s12882-025-04118-y (PMC12080063; doi:10.1186/s12882-025-04118-y)
Supplement: Supplementary file 3 — Supplementary Material 3 [file 12882_2025_4118_MOESM3_ESM.docx]

**Supplemental Figure 1: Pre/Post Pregnancy Lab Value Changes**

**Supplemental Figure 1:** **:** Changes in lab values pre-post pregnancy (during/after C3G presentation). The differences in all pre/post lab values were evaluated using median difference and p-values from Wilcoxon matched-pairs signed rank tests, represented by asterisks. Pre/post-pregnancy lab values are within 3-6 months of pregnancy.

| **Supplemental Table 1: Changes in C3G+P Clinical Lab Values of Renal Significance Pre/Post Pregnancy** | | | | |
| --- | --- | --- | --- | --- |
| **Clinical Lab** | **Pre-Pregnancy Median (IQR)** | **Post-Pregnancy Median (IQR)** | **Median Post - Pre Difference (IQR)** | **P value** |
| sCr (mg/dL) | 0.76 (0.63 – 1.00) | 0.81 (0.67 – 1.22) | 0.09 (-0.01 – 0.15) | 0.02 |
| eGFR (ml/min) | 92 (66 - 113) | 86 (54 - 105) | -6 (-20 - 1) | 0.04 |
| Systolic BP (mmHg) | 128 (114 - 132) | 135 (123 - 145) | 2 (-10 - 31) | 0.10 |
| Diastolic BP (mmHg) | 80 (72 - 89) | 82 (76 - 88) | 2 (-6 - 12) | 0.43 |
| UA Protein | 2+ (1 - 2+) | 2+ (2 - 3+) | 1+ (0 - 1+) | 0.01 |
| Urine Protein/  Creatinine Ratio (g/g) | 1.40 (0.83 – 3.66) | 2.80 (1.44 – 3.89) | 0.45 (-0.06 – 2.07) | 0.02 |
| Weight (kg) | 68.5 (52.3 – 120.2) | 75.1 (56.0 – 102.0) | 2.7 (-20.4 – 25.0) | 0.18 |
| Complement C3 (mg/dL) | 89 (36 - 130) | 89 (23 - 139) | -9 (-17 - 11) | 0.50 |

**Supplemental Table 1:** Changes in C3G+P selected lab values before and after pregnancy. Differences are the pre-pregnancy median value subtracted from the post-pregnancy median value. P values were calculated using Wilcoxon’s test. Pre/post-pregnancy lab values are within 3-6 months of pregnancy.

| **Supplemental Table 2: Reported Pathogenic Variants and Variants of Unknown Significance** | | | | | | | | |
| --- | --- | --- | --- | --- | --- | --- | --- | --- |
| **Patient** | **Gene Name** | **Chromosomal Location** | **Coding Position** | **Protein Position** | **Zygosity** | **CADD Score** [[40]](https://doi.org/10.1093/nar/gkad989) | **Interpretation** | **MLPA** |
| **1** | ***C3*** | **19:6702188:T>A** | **c.2390A>T** | **p.Asp797Val** | **Het** | **28** | **VUS** | **Negative** |
|  | ***CFI*** | **4:110663642:C>A** | **c.1534+5G>T** | **splice_region_variant, intron_variant +** | **Het** | **21.8** | **VUS** |  |
|  | ***PLG*** | **6:161152905:C>T** | **c.1567C>T** | **p.Arg523Trp** | **Het** | **19.54** | **VUS** |  |
| **2** | **NEGATIVE** | | | | | | | **Negative** |
| **3** | ***CFH*** | **1:196709816:G>T** | **c.2850G>T** | **p.Gln950His** | **Het** | **13.7** | **VUS** | **Heterozygous deletion of CFHR3-CFHR1 (Common)** |
| **4** | **NEGATIVE** | | | | | | | **Negative** |
| **5** | **NEGATIVE** | | | | | | | **Negative** |
| **6** | ***THBD*** | **20:23029222:G>A** | **c.920C>T** | **p.Ser307Leu** | **Het** | **13.9** | **VUS** | **Negative** |
| **7** | **NEGATIVE** | | | | | | | **Heterozygous deletion of the genes CFHR3-CFHR1 (Common)** |
| **8** | ***CFH*** | **1:196659231:C>A** | **c.1198C>A** | **p.Gln400Lys** | **Het** | **0.02** | **VUS** | **Negative** |
| **9** | ***CFHR2*** | **1:196918738:C>T** | **c.212C>T** | **p.Thr71Met** | **Het** | **1.6** | **VUS** | **Homozygosity for del(CFHR3-CFHR1)** |
| **10** | **NEGATIVE** | | | | | | | **Negative** |
| **11** | **NEGATIVE** | | | | | | | **Negative** |
| **12** | **NEGATIVE** | | | | | | | **Three copies of *CFHR3-CFHR1*** |
| **13** | **NEGATIVE** | | | | | | | **Heterozygous deletion of the genes CFHR3-CFHR1 (Common)** |
| **14** | ***CFH*** | **1:g.196648827:C>T** | **c.694C>T** | **p.Arg232Ter** | **Het** | **34** | **Pathogenic** | **Negative** |
| **15** | **NEGATIVE** | | | | | | | **Not Available** |
| **16** | ***ADAMTS13*** | **9:136307538:G>A** | **c.1987G>A** | **p.Glu663Lys** | **Het** | **14.2** | **VUS** | **Negative** |
| **17** | **NEGATIVE** | | | | | | | **Not Available** |
| **18** | **NEGATIVE** | | | | | | | **Heterozygous deletion of the genes CFHR3-CFHR1 (Common)** |
| **19** | **NEGATIVE** | | | | | | | **Heterozygous deletion of the genes CFHR3-CFHR1 (Common)** |
| **20** | **NEGATIVE** | | | | | | | **Negative** |
| **21** | ***PLG*** | **6:161173155:G>A** | **c.2134G>A** | **p.Gly712Arg** | **Het** | **22.3** | **VUS** | **Fusion of CFHR5 scr 1-2 & CFHR2 scr 1-4 (Pathogenic)** |
| **22** | **NEGATIVE** | | | | | | | **Negative** |
| **23** | **NEGATIVE** | | | | | | | **Negative** |
| **24** | **NEGATIVE** | | | | | | | **allele 1 homozygous del(CFHR3-CFHR1), allele 2 dup(CFHR1-CFHR4)** |
| **25** | ***ADAMTS13*** | **9:136305591:C>T** | **c.1913C>T** | **p.Pro638Leu** | **Het** | **24.8** | **VUS** | **Heterozygous deletion of the genes CFHR3-CFHR1 (Common)** |
| **26** | **NEGATIVE** | | | | | | | **Negative** |
| **27** | ***C3*** | **19:6718304:G>C** | **c.387C>G** | **p.Tyr129Stop** | **Het** | **35** | **VUS** | **Negative** |
|  | ***THBD*** | **20:23028640:G>A** | **c.1502C>T** | **p.Pro501Leu** | **Het** | **19.9** | **VUS** |  |
| **28** | **NEGATIVE** | | | | | | | **Negative** |
| **29** | **Not Available** | | | | | | | **Not Available** |
| **30** | **NEGATIVE** | | | | | | | **Negative** |
| **31** | **NEGATIVE** | | | | | | | **Negative** |
| **32** | ***THBD*** | **20:23028528:G>T** | **c.1614C>A** | **p.His538Gln** | **Het** | **24.1** | **VUS** | **Not Available** |

**Supplemental Table 2:** Genetic variants identified in patients within the cohort. Testing was completed by the Molecular Otolaryngology and Renal Research Laboratory with the Genetic Renal Panel v8. Included genes are as follows: *CFH, CFI, MCP, CFB, CFHR5, THBD, C3, ADAMTS13, PLG, DGKE, MMACHC, G6PD, WT1, C5.* CADD Scores were calculated with GRCh37-v1.7 [[40]](https://doi.org/10.1093/nar/gkad989). MLPA (multiplex ligation-dependent probe amplification) copy number variation screening was performed only for the CFH-CFHR5 genomic region. For more information, see <https://morl.lab.uiowa.edu/clinical-diagnostic-services/clinical-renal-diagnostic-division/genetic-renal-panel-testing>.

| **Supplemental Table 3: Adverse Health Outcomes in Neonate Descriptions** | | |
| --- | --- | --- |
|  | **Maternal Description** | **Neonate Description** |
| Mother #1 – Driver = C3 and C5 Nephritic Factors | Diagnosed 8 years prior to pregnancy, frequent urinary tract infections, developed preeclampsia, was started on methyldopa, UPC steadily advanced throughout pregnancy peaking at 5.1 with normal sCr. | 36w 3d – decreased movement with HR 160, infant delivered by emergency cesarean section at for fetal distress, postnatal finding of stroke, borderline intra-uterine growth restriction, some delayed milestones but no long-term issues. |
|  | 4 years after first pregnancy, preemptive switch to methyldopa with subsequent rise in UPC, normal sCr, worsening HELLP labs at 25w treated w/ labetalol, severe range BP and preeclampsia at 26w, severe edema for 2 weeks postpartum. | 26w 2d- Required emergency cesarean section d/t preeclampsia and decreased fetal movement, intra-uterine growth restriction, hospitalized for approx. 4 months, but no long-term issues. |
| Mother #2 –  Driver = Monoclonal Immunoglobulin | Diagnosed 1 year prior to pregnancy, normal pregnancy course with stable sCr, proteinuria peaked at 1.9 g/d with stable BP. | 39w 0d- normal immediately after delivery, admitted 1 week later with acute renal failure, sCr 1.6 mg/dL, discovered monoclonal immunoglobulin transfer from mother. Issue resolved with double volume exchange transfusion and no long term issues. |
| Mother #3 –  Driver = Unknown | Diagnosed 17 years prior to pregnancy, rising sCr and BP’s throughout pregnancy, added Labetalol and Procardia, BTMZ injections at 30/34 wks, preeclampsia at 34 wks. | 36w 2d- induced labor d/t preeclampsia, intrauterine growth restriction 5^th^ percentile, some cognitive delays but no other long-term issues. |
| Mother #4 –  Driver = C4 and C5 Nephritic Factors | Diagnosed 15 years prior to pregnancy, Cardizem for BP, preeclampsia at 35 wks with AKI during induction, sCr 0.77 -> 1.33, resolved to 0.98 1 week post-delivery. | 36w 0d- induced labor d/t preeclampsia, pneumothorax requiring feeding tube and O2, resolved spontaneously with discharge at 10 days, 18^th^ growth percentile at 6 months but no long term issues. |

**Supplemental Table 3:** Descriptions of the pregnancies experiencing complications of the neonate/NICU Hospitalization, as well as maternal renal disease course.


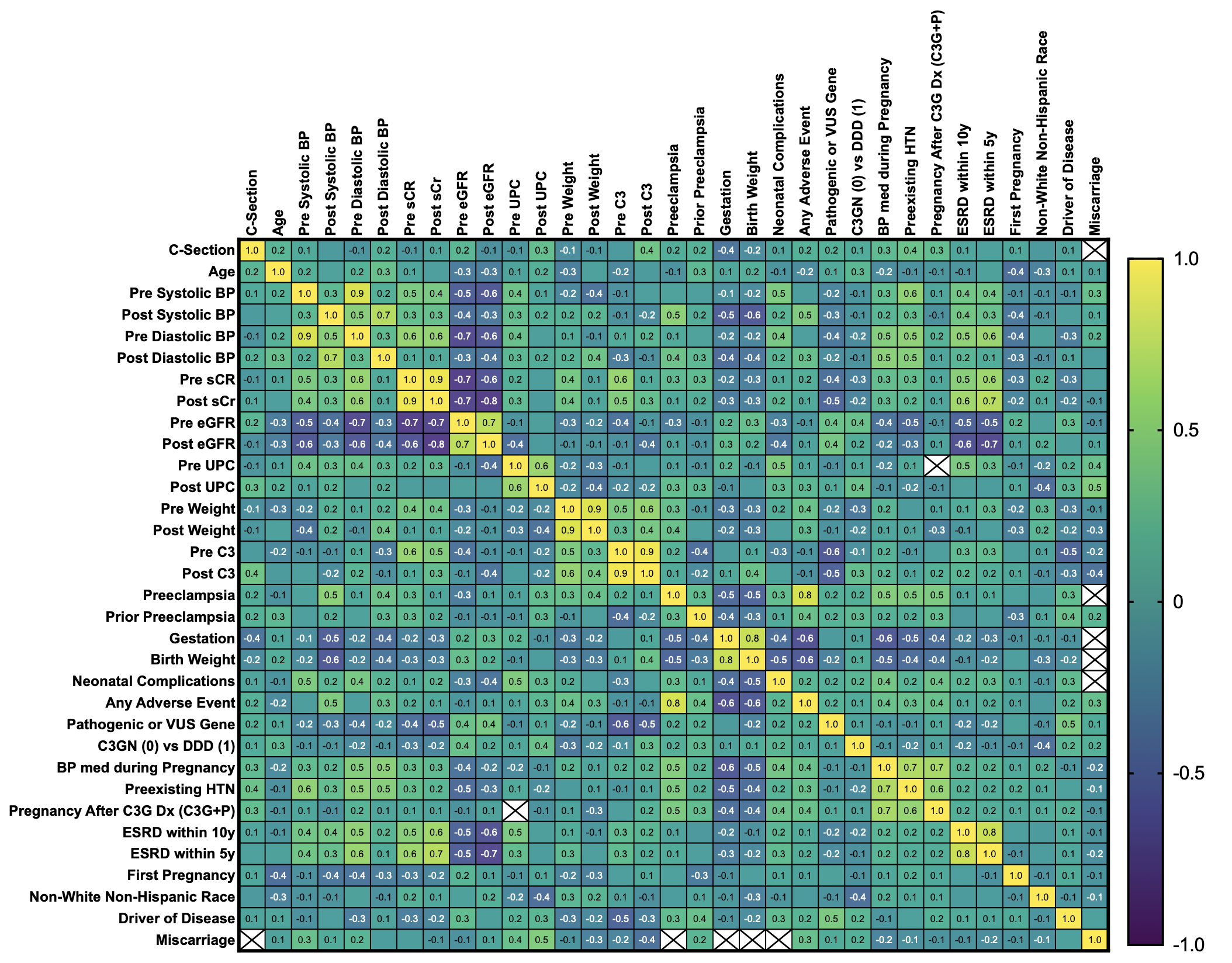


**Supplemental Figure 2: Correlation Matrix Heatmap**

**Supplemental Figure 2:** Labels in each box represent correlation coefficient r. Calculated using nonparametric Spearman’s correlation. White boxes with an X represent correlations with n <3 pairs or the comparison of exclusive events. Pink boxes with no correlation coefficient r fall between -0.1 and 0.1. Discrete variables were coded 1/0 for presence or absence of listed event or characteristic.
